# Community engagement strategies improve recruitment and enrollment in a pragmatic clinical trial

**DOI:** 10.1017/cts.2025.10103

**Published:** 2025-07-24

**Authors:** Kari G. Vance, Jonah Pedelty, Barbara J. Van Gorp, Carol G.T. Vance, Elizabeth M. Johnson, Fangfang Jiang, David-Erick Lafontant, Maxine Koepp, Andrew A. Post, Emine Bayman, Ruth L. Chimenti, Dana L. Dailey, Leslie J. Crofford, Heather Reisinger, Kathleen A. Sluka

**Affiliations:** 1 University of Iowa Roy J and Lucille A Carver College of Medicine, Department of Physical Therapy & Rehabilitation Science, Iowa, IA, US; 2 Vanderbilt University Medical Center, Department of Rheumatology, Nashville, TN, US; 3 University of Iowa College of Public Health, Department of Biostatistics, Iowa, IA, US; 4 St Ambrose University, Physical Therapy Department, Davenport, IA, US; 5 University of Iowa, Department of Internal Medicine, Iowa, IA, US

**Keywords:** Community engagement, pragmatic trial, physical therapy, pain, recruitment, enrollment

## Abstract

**Introduction:**

Rural communities make up 19% of the US population, yet are underrepresented in clinical trials. Community engagement methods can facilitate collaboration and trust with local healthcare personnel to enhance enrollment. The purpose of this manuscript is to describe community engagement methods and their impact on enrollment in a pragmatic clinical trial.

**Methods:**

We describe a variety of methods used in the Fibromyalgia TENS in Physical Therapy Study (FM-TIPS) to enhance enrollment in rural communities and low-enrolling clinics. Community engagement methods were implemented partway through the trial for selected groups: Targeted Rural (TR) (*n* = 10), Targeted Low Enrolling (TLE) (*n* = 6), and compared to Untargeted Groups (UT) (*n* = 13). The impact of these methods on inquiries, screening, and enrollment were evaluated by comparing actual enrollment to projected enrollment.

**Results:**

We trained and employed community engagement coordinators to implement strategies in TR and TLE physical therapy clinics. These included, posting flyers, community events, physician outreach, social media ads, and direct mailing. These methods increased study inquiries, screening and enrollment in the study. Specifically, when compared to projected values there were increases in enrollment for both the TR and the TLE groups, but not the UT group. Of those that passed screening 99% of rural and 32% of urban residents enrolled in the study.

**Conclusion:**

A multi-pronged and individualized community engagement approach can increase enrollment of rural residents in clinical trials. Building strong relationships and partnering with community clinics and local communities is essential to success.

## Introduction

Chronic pain is a major health problem affecting 20% of U.S. adults, with 7–8% experiencing pain severe enough to impair daily function [1,2]. Social determinants of health – including education, income, geographic location, race, and ethnicity – influence both the prevalence and impact of chronic pain [1,3–7]. Particularly relevant to the current study is the greater burden of chronic pain among rural populations exacerbated by reduced access to evidence-based pain management [4,8–11]. Specifically, rural populations experience higher rates of pain and more severe pain compared to urban populations [5,6], and limited access to healthcare providers and pain specialists contributes to worse outcomes [8–10,12]. For instance, rural residents with low back pain experience greater functional limitations, depression, and disability than their urban counterparts [13]. Thus, clinical studies that include rural residents could provide unique insights into pain and its management.

A key factor contributing to healthcare inequities in rural communities is underrepresentation of rural populations in clinical research. Clinical trials are predominately conducted in academic medical centers in urban locations and it is unclear how many rural participants are included in these trials [14–20]. Rural communities make up 19% of the US population, and this lack of inclusion reduces generalizability of clinical research findings [4,8–11,14,21]. Conducting clinical trials in rural settings presents unique challenges, many of which stem from historical disinvestment in rural healthcare infrastructure and mistrust fueled by the opioid crisis [8,22–25]. Rural residents with chronic pain are more likely to be prescribed opioids and less likely to use non-pharmacological treatments including physical therapy [5,24,26,27], despite strong evidence and clinical guidelines supporting non-pharmaceutical treatments as first line treatments [7,28–30]. Furthermore, awareness of clinical trials remains low among both patients and healthcare providers in rural areas, posing significant challenges to recruitment and enrollment [14,22,31–33]. Collaboration with trusted local healthcare personnel to develop a multi-faceted approach to recruitment is a primary facilitator to clinical trial participation in rural communities [34–38]. Pragmatic clinical trials, which take place in real-world healthcare settings, provide a valuable opportunity to establish partnerships with local healthcare providers.

Fibromyalgia is a chronic pain condition characterized by chronic widespread pain and fatigue where physical therapy is a primary treatment [28,29]. The Fibromyalgia TENS in Physical Therapy Study (FM-TIPS) is a pragmatic clinical trial conducted in outpatient physical therapy clinics located in both rural and urban settings (39). The purpose of this manuscript is to describe implementation of community engagement strategies in a pragmatic trial and their impact on enrollment for a targeted rural group of clinics (primary aim) and a targeted low-enrolling group of clinics (secondary aim).

## Methods

### Study design

The primary aim of FM-TIPS was to determine if the addition of Transcutaneous Electrical Nerve Stimulation (TENS) to routine physical therapy improves movement-evoked pain for individuals with fibromyalgia [39]. FM-TIPS was approved by the Human Subjects Review Board at the University of Iowa. FM-TIPS was a cluster-randomized pragmatic clinical trial (NCT04683042) conducted in 28 outpatient physical therapy clinics spanning six healthcare systems (HCS) and seven states in the Midwest with a recruitment goal of 450. FM-TIPS recruited from 15 urban and 13 rural clinics that were defined based on Rural-Urban Commuting Areas (RUCA) codes (Economic Research Service, 2022). Recruitment began in February 2021 and finished in September 2024. Physical therapists at each enrolling clinic received IRB training, protocol training, and intervention (TENS) training prior to initiation of recruitment. Potential participants were identified by participating physical therapists at their initial visit based on defined inclusion and exclusion criteria: diagnosis of fibromyalgia and no contraindications to TENS. Ongoing recruitment strategies were directed to the participating clinics (Table 1). Each participating physical therapist was trained in human subject research and study procedures. Each clinic was assigned a separate study team liaison who communicated regularly with the clinic through monthly meetings either on-site or virtually, text, and email to directly interact with enrolling physical therapists, answer questions, discuss study updates, and review enrollment progress. FM-TIPS also included a schedule of non-monetary gifts and prizes to local clinics for reaching recruitment milestones, screening, and enrollment competitions between clinics within a healthcare system, flyers and materials placed in each clinic, and personalized letters to referring physicians. These strategies were initiated after initial enrollment in February 2021 and continued until the end of recruitment in September 2024.

**Table 1.** Ongoing recruitment and community engagement efforts. A variety of recruitment efforts were implemented in all clinics beginning at the time of first enrollment. These efforts are those that occur beyond initial training of enrolling physical therapists (enrolling PTs). The majority of methods prior to implementation of community engagement were directed to the healthcare system (HCS), clinics and physical therapists. Recruitment was an iterative process throughout the study and a number of different methods were added throughout the period. Community engagement methods were primarily directed to community members which included potential participants and referring providers. Again, this was an iterative process throughout the 16 months of community engagement. Initial dates of the first instance of a method are given, but once considered successful these methods were ongoing through the end of enrollment in September 2024. Recruitment and community engagement strategies, *physician liaison from HCS scheduled one community education event during the training phase of community engagement coordinator

Recruitment Strategies (all clinics) February 2021-September 2024	Stakeholders	Date Implemented
In-clinic flyers	Patients at participating clinics	February 2021
Enrollment milestone incentives (non-monetary)	Enrolling PTs	February 2021
Virtual and on-site visits from study team liaison	Enrolling PTs	February 2021
Weekly enrollment reports	Enrolling PTs, HCS	February 2021
Monthly newsletter	Enrolling PTs, HCS	February 2021
Immediate help contact number for study team	Enrolling PTs, HCS	February 2021
Monetary reimbursement for enrollment	Clinic/HCS	February 2021
Quarterly meetings with HCS liaisons	HCS	February 2021
Tips sheet and study protocol book	Enrolling PTs	February 2021
Website with handbook, education materials, and contact information	Enrolling PTs	February 2021
Provider letters	Local medical providers	June 2021
Promotional materials for clinic websites	Clinic/HCS	December 2021
Continuing Education	Enrolling PTs	February 2022
Enrollment and screening contests by HCS	Enrolling PTs	January 2023
Personalized recruitment goals	Clinic/HCS	May 2023
**CE Strategies (targeted clinics)** June 2023-September 2024		
Community Education Events	Community Members	April 2023*
Community flyers	Community Members	June 2023
In-person provider outreach	Local Medical Providers	June 2023
Media Outreach	Community Members	August 2023
Direct Mail	Community Members	October 2023
Website updates	Community Members	January 2024
Facebook Ads	Community Members	April 2024
Google Ads	Community Members	June 2024

Partway through enrollment, the study was awarded a diversity supplement to enhance enrollment using community engagement methods with a primary focus on rural participants. This allowed us to examine the impact of community engagement strategies before and after implementation. Based on rurality code, current recruitment status, and input from each HCS regarding individual clinic’s capacity to handle increased enrollment, clinics were divided into three groups as follows: Targeted Rural (TR) (*n* = 10; RUCA 4–10), Targeted Low Enrollment (TLE) (*n* = 6; RUCA 1–4), and Untargeted (UT) (*n* = 12; RUCA 1–7). Two clinics in the UT group were rural – one was deactivated, and one was enrolling over target. The TR group were 1) located in communities with a RUCA code of 4–10, 2) currently enrolling participants at a rate commensurate with recruitment goals, and 3) had the capacity to enroll a greater number of participants. The TLE group were not meeting recruitment goals, as defined by <5 total patients enrolled or no enrollments over the previous six months. Lastly, we maintained existing recruitment strategies and did not implement community engagement strategies for 12 clinics that were meeting or exceeding recruitment goals, termed untargeted group. Community engagement strategies were initiated in June 2023 into the TR and TLE groups. Thus, the aim of this project was to describe these newly implemented CE strategies and their impact on screening and enrollment using a pre- and post-design.

### Community engagement recruitment strategies

Two full-time Community Engagement (CE) Coordinators led the community engagement initiatives. Unique strategies were applied to each clinic and surrounding communities depending on community research and observations by the CE Coordinators, and feedback from the physical therapy clinic staff. The CE coordinators were trained and directed by an anthropologist who used an ethnographic perspective and had expertise in community engagement methodology. The CE coordinators researched each community by familiarizing themselves with demographics, major employers, local organizations and clubs, and local events. In addition, the coordinators identified major medical providers in the area, disability assistance programs, churches, and extension services.

The initial research was followed by interviews with the physical therapists at each of the TR and TLE clinics and study team liaisons who served as the main point of contact for the clinics. These interviews gathered the clinician’s perceptions of the community and its relationship to health and well-being. Additionally, physical therapists provided opinions on recruitment strategies for the CE coordinators to implement. Two of the six healthcare systems employed physician liaisons with whom the CE coordinators worked to advertise the study and to set up educational events in the local communities. Meetings were held with study team liaisons to get their impressions of the enrolling PTs and clinics at each location. These meetings informed development of tailored community engagement plans for each clinic using a multi-pronged approach.

Formal community engagement plans involved establishing relationships with clinics, physical therapists who enrolled patients, towns, and surrounding communities by directly visiting local communities. The frequency of community engagement visits ranged from every few weeks to quarterly and included posting flyers in public spaces; hosting, attending, or participating in community events; and visiting local physicians. We also promoted study awareness through direct mailing, online advertisements, and radio and newspaper stories.

Revisions were made to the study website in January 2024 to better meet the needs of potential participants and based on input from study staff. Initially, the website was directed toward participating physical therapists and provided them with resources to assist with recruitment and enrollment. The revisions were part of our community engagement strategy and included changing the home page to include basic eligibility requirements, a short list of study activities, and a semi-interactive map of all clinic locations. Interested potential participants were directed to fill out a contact form on the study website which routed to the study team email.

### Data analysis

All inquiries by potential participants received a contact from a study team member, primarily by phone. Participants were given study details that included enrollment criteria, aims, and study requirements. All interactions and subsequent actions taken at a clinic, including whether the participants screened and successfully enrolled, were tracked on the study inquiry log both before and after community engagement methods were collected.

Baseline demographics data were collected from participants who met the modified intention-to-treat criteria, defined as any participant who provided data to the study. These data were recorded after enrollment during the first study visit. Data are summarized as means, standard deviations, and range, for continuous variables or number of samples and percentages for categorial variables. To test for differences between the three groups (TR, TLE, and UT) data were compared using analysis of variance for continuous variables, and Chi-squared tests or Fisher’s exact tests for categorical variables. To assess the impact of community engagement on inquiries, screening, and enrollment, we compared projected vs actual numbers over the 16 months of community engagement using a paired *t*-test. Projections were estimated based on average monthly enrollment rate prior to implementation of community engagement and compared to actual enrollment across individual groups.

## Results

### Clinics

**Figure 1.**
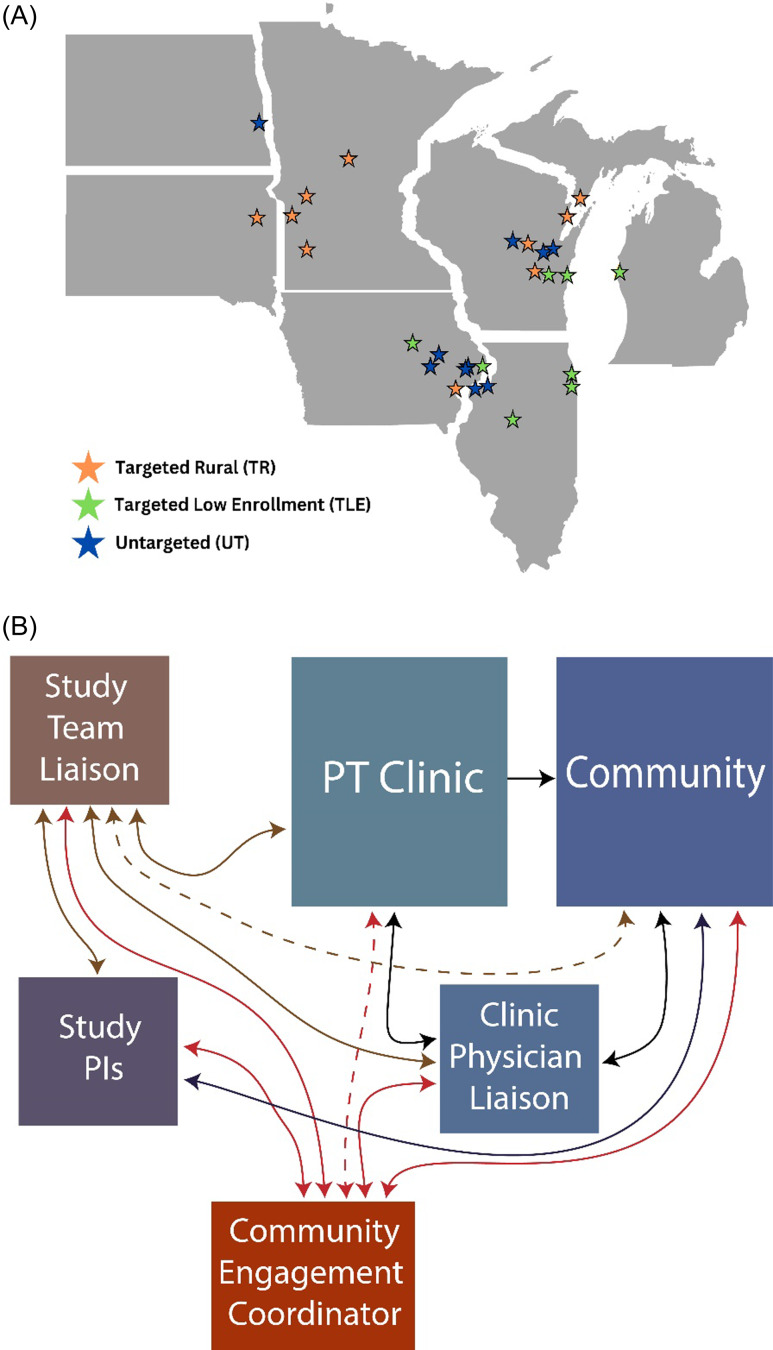
Clinic distribution and study team interactions. (A) Map showing the location of clinics across the Midwest by group – targeted rural (TR), targeted low-enrolling (TLE), untargeted (UT). (B) Diagram showing the study team interactions with the PT clinics and the community. The community engagement coordinators interacted with all other members of the study team, PT clinics, physician liaisons, and the community. Their primary role was to enhance engagement in the community, and they utilized help from the study team liaisons for their expertise on the clinics and physical therapy. Study team liaisons also interacted with all members of the team, PT community, physician liaisons and the community. Their primary role was to interact and engage the PT clinics and they provided PT-related community talks and expertise on outreach materials. Study PIs interacted primarily with the study team liaison’s and community engagement coordinators, but also attended meetings with the PT clinics with the study team liaisons, and tried to interact with large health care systems in the communities. Dotted lines represent a supporting role, while solid lines represent primary interactions.

Of the 28 clinics in the study (Figure 1a), 46% were located in a rural setting and 71% enrolled rural residents. Two urban clinics were located in a major metropolitan area. The other 10 urban clinics were in communities with populations ranging from 6,726 to 192,648.

The study population had a mean age of 53.2 years and was predominantly female (91%), White (82%), and non-Hispanic (85%). Differences between groups were observed for age, marital status, race and ethnicity, and education (Supplemental Table 1). Interestingly, all 3 groups traveled the same distance to physical therapy clinics (8.6–9.4 miles).

### Community engagement strategies

**Figure 2.**
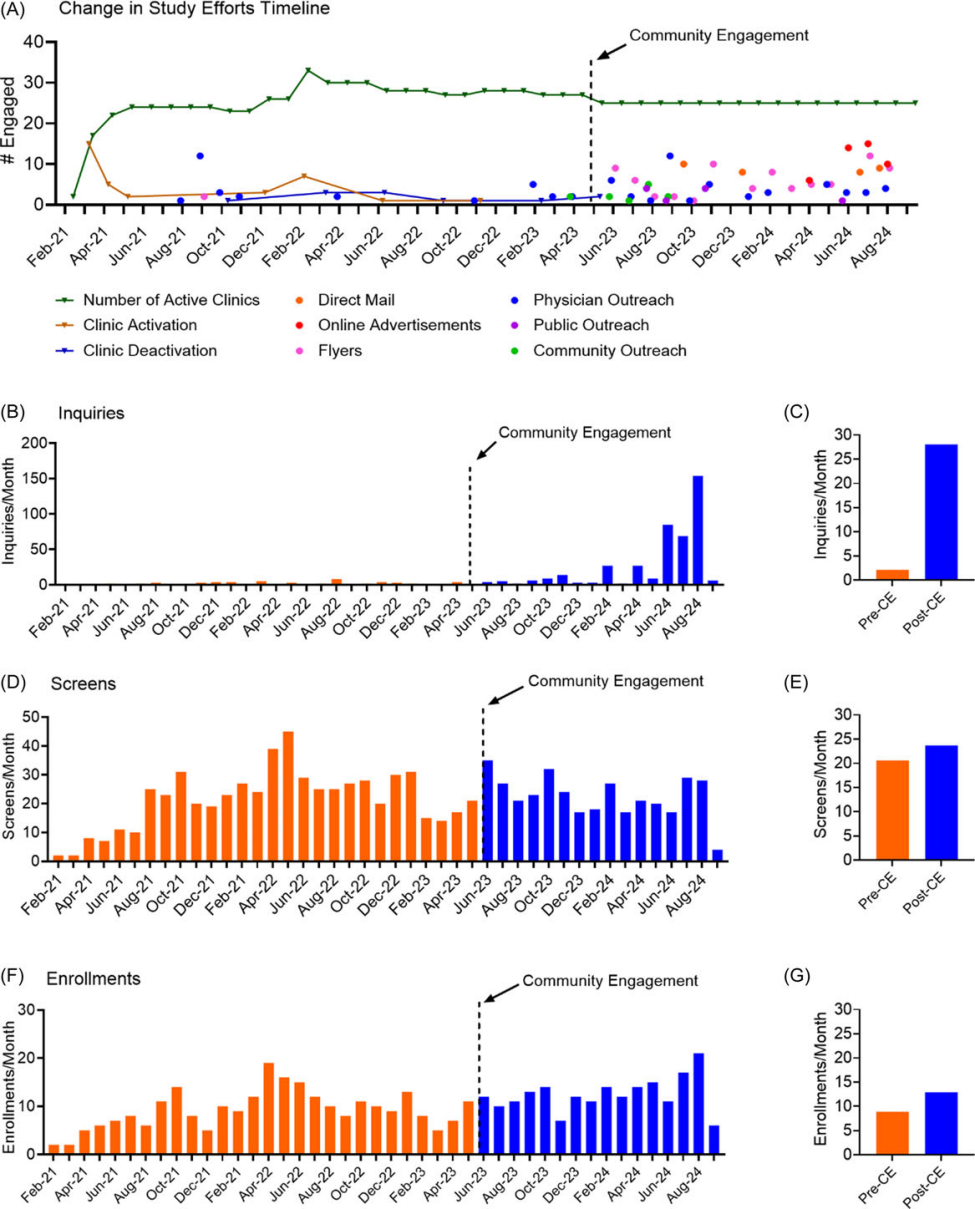
Inquiries, screens, and enrollments per month. **(**A) timeline of implementation. Each colored line represents the total number of clinics that were active (green), when clinics were activated (orange), or deactivated (blue) during the study. Each circle represents the number of clinics engaged and the month and year a community engagement effort that was done. (B) timeline graph of inquiries across the study timeline. (C) bar graph shows the average number of inquiries per month before and after community engagement efforts. (D) timeline graph of screens across the study timeline. (E) bar graph shows the average number of screens per month before and after community engagement efforts. (F) timeline graph of enrollments across the study timeline.(G) bar graph shows the average number of enrollments per month before and after community engagement efforts. Dotted lines on the timeline graphs indicate the date of implementation of community engagement methods. CE = community engagement.

The study team included multiple groups that routinely interacted with each other, the clinics, and the community to implement ongoing recruitment and community engagement strategies (Figure 1b). Here we describe those activities specific to community engagement. Community engagement coordinators conducted 108 community visits among 20 communities which included visits to community sites as well as short visits to the physical therapy clinic serving that community to provide food incentives and updated study materials (Table 1). Additional methods included direct mailing to potential participants, social media ads, and local media outreach. These methods were distributed across 16 months after implementation (Figure 2a). Of note, all the physical therapists and clinics were located within the community they served and partnered with study team to develop community directed materials and methods.

***Flyers***: Flyers were developed for each community to connect with individuals who visit community centers and local businesses. Study team members with fibromyalgia, community engagement coordinators, study team liaisons, and clinic representatives all participated in development of flyers. Flyers included eligibility criteria, potential benefits of participating, and the local physical therapy clinic’s logo and location in addition to information for contacting the study team. Each healthcare system was offered clinic-specific modifications to include the contact information based on clinician preference (Supplemental Figure 1a). Community locations for flyers were selected through in-person and online research for each community and included community centers, libraries, gyms, chiropractic and dental offices, and local businesses if they had a community bulletin board. Community engagement coordinators checked locations periodically to ensure flyers remained posted or to replace flyers.

Flyers were posted in approximately 400 locations across 16 communities between June 2023 and August 2024 (Supplemental Table 2). Costs included printing and travel expenses to communities for flyer distribution. Travel distance ranged from 1–8 hour drives sometimes requiring overnight stays. Multiple community visits were completed within a single trip to maximize time and cost efficiency.

**Potential referring providers:** To increase awareness of the study among local healthcare providers, potential referring providers were contacted by the study team and/or the clinic’s physical therapists. Providers included local primary care physicians, rheumatologists, pain specialists, dentists, massage therapists, chiropractors, and wellness centers. Prior to community engagement, 515 letters were sent to nearly all communities based primarily on online searches of potential referring providers. After implementation of community engagement, we shifted strategies consulting with clinics and physician liaisons first to suggest and confirm where to send letters, and to develop and deliver study packets to local referring providers. After implementation of community engagement methods, we sent 237 letters and delivered 69 packets of materials to referring providers associated with 10 clinics. The study team liaisons, who were physical therapists, dropped off flyers and study information to front desk staff and physicians during their on-site clinic visits. Attempts to schedule meetings with providers were made and ultimately unsuccessful. We were also unsuccessful in contacting healthcare systems, outside of participating physical therapy healthcare systems, to increase referrals to the study clinics.

**Community education events:** To increase awareness of evidence-based chronic pain management, a variety of community events were attended including ice cream socials, a community walk for opioid awareness, farmer’s markets, and educational sessions held at local libraries, YMCAs, and within clinics. Partnerships between local physical therapists, healthcare system physician liaisons, community engagement coordinators, and study team liaisons facilitated educational events. Educational sessions addressed benefits and basic methods of physical therapy for chronic pain management. Educational sessions were presented by a study team liaison physical therapist in collaboration with a local clinic physical therapist. During the training phase for the community engagement coordinators (April 2023), one community education event was scheduled by the physician liaison from one HCS, and thus, one event occurred before the June 2023 implementation for the rest of the community engagement strategies.

The study team organized or attended nine community events. Most events were attended by 4–12 people, though some had zero attendance. The community events did not appear to have a direct impact on inquiries, screening, or enrollment. However, community events raised awareness within the community for research, fibromyalgia and chronic pain, and the clinic itself. It also showed our commitment and support to the clinics and served to keep physical therapists engaged in study activities.

**Direct mail:** To ensure we connected with community members that may have limited access to public spaces or healthcare providers a large postcard (5x7”) was developed to advertise the study and was individualized to each clinic (Supplemental Figure 1B). The United States Postal Service’s (USPS) Every Door Direct Mail service was used to identify households. The USPS direct mail program uses census data to report average age and income on each route. Routes were prioritized by the greatest percentage of inhabitants with ages similar to study participants. In several instances for TR clinics every mail route in the community was selected. Other considerations included proximity to the clinic and local clinician recommendations. Postcards directed interested parties to contact the clinic or the study team by email or telephone.

Direct mail campaigns were implemented four separate times throughout the recruitment period (Table 2). The four direct mail campaigns sent a total of 169,127 recruitment postcards which resulted in 127 potential participant inquiries to the study. Clinics also reported increased inquiries, screening and enrollment in response to postcards. A few clinics reported that the increased inquiries and need for screening was overwhelming. Later campaigns removed the option to contact the clinic and were directed to the study team through the website, email or phone. The average cost was $334.4 per 1000 postcards, including printing, university mail services, and postage (Table 2).

**Table 2.** Campaigns for direct mail outreach. Table shows the dates, number of clinics targeted, metrics and general costs for direct mail. TR = targeted rural; TLE = targeted low enrolling

Direct Mail Date	Clinic Target Status	# of Clinics	# of Mail Pieces	Total Mail Pieces	Total Cost	$ per 1000
Oct-23	TR	10	14530	14,530	$4,742	$326
	TLE	0	0			
Jan-24	TR	5	4865	9,530	$3,463	$363
	TLE	3	4665			
Jun-24	TR	4	18705	46,815	$15,349	$328
	TLE	4	28110			
Aug-24	TR	5	40384	98,252	$31,413	$320
	TLE	4	57868			

***Online advertisements:** advertisements were targeted to potential participants by proximity to the clinic and clinician recommendations (Supplemental Figure 1C). A radius around each clinic was determined based on travel time to the clinic and recommendations from physical therapists in each clinic which often included neighboring communities beyond the initial radius. Facebook advertisements directed interested parties to contact the study team through our website the study also implemented advertisements through google search which was set up to include the FM-TIPS website as a sponsored listing in google search and targeted communities by location.*

The Facebook ad campaigns resulted in targeted recruitment advertising running for 64 days (Table 3). The campaigns resulted in 164 potential participant inquiries to the study team. Facebook advertisements were budgeted for $13,240 and actual costs were $11,359. The ad was shown on screen over 2.5 million times with an average cost of $4.41 per 1000 impressions. The Google ads campaigns resulted in targeted recruitment advertising running for 60 days. No study inquiries were traceable to Google ads, however, an increase in traffic to our website in July 2024 was traceable to Google advertisements. Google advertisements were budgeted for $6,600 and actual costs were $4,557. The advertisement was shown on screen over 1.3 million times with an average cost of $4.57 per 1000 impressions (Table 3).

**Table 3.** Campaigns for social media outreach. Table shows the dates, number of clinics targeted, metrics and general costs for facebook and google ads. TR = targeted rural; TLE = targeted low enrolling; UT = untargeted

	Start Date	Clinic Target Status	# of Clinics	Duration	Budget/Day/Clinic	Budget	Actual	Impressions	Cost/1000 Impressions	Link Clicks	Cost Per Click
**Facebook**	April-24	TR	3	14 days	$10	$840	$840	153,582	$5.47	1,066	$0.79
		TLE	3								
		UT	0								
	Jun-24	TR	6	30 days	$20	$8,400	$8,400	1,801,133	$4.32	10,810	$0.72
		TLE	5								
		UT	3								
	Aug-24	TR	5	20 days	$20	$4,000	$4,060	621,985	$4.42	3,814	$0.72
		TLE	4								
		UT	1								
**Google**	Jun-24	TR	6	30 days	$20	$3,600	$3,287	1,162,003	$2.83	7,327	$0.45
		TLE	0								
		UT	0								
	Jul-24	TR	3	30 days	$20	$3,000	$1,270	201,242	$6.31	1,763	$0.72
		TLE	1								
		UT	1								

**Media outreach:** Area newspapers and local community newsletters were targeted to increase exposure and awareness of the study around each community. The CE coordinators wrote short articles highlighting the study and the participating physical therapy clinic. Topics included the current opioid crisis associated with chronic pain, stories about the FM-TIPS study, and the clinics participating in the local community. Stories were distributed to 14 news outlets, but only 3 news sources published our stories.

Additional efforts to disseminate information about the study included attempts to do radio interviews. With the help of a physician liaison, a study team liaison completed one radio interview with a station in listening radius of several clinics. We also developed a Facebook page and posted educational information about the study, chronic pain, fibromyalgia and physical therapy management.

Three rural clinics were eligible to be nominated for an award from their state’s Office of Rural Health based on their contributions to research and commitment to improving the health of their communities. The study team nominated these three eligible clinics, and all three clinics received the award and were highlighted in local newspapers.

### Effects of community engagement

**Study inquiries:** From the initiation of community engagement strategies in June 2023 to August 2024, the study team documented 425 inquiries (Figure 2b). Potential participants contacted the study team after learning about the study from a variety of methods. Of those 425 inquiries, 164 came from Facebook Ads, 127 from direct mailing, 41 from flyers, and 11 from physician referral (Table 4). Of the 425 inquiries, 27 individuals screened and 25 enrolled. Average monthly inquiries across all clinics increased from 2.15 inquiries per month prior to community engagement to 28.07 inquiries per month after implementation of community engagement strategies (Figure 2c). Of the 238 individuals willing to share their zip codes (April 2024-August 2024), 118 were rural and 120 were urban.

**Table 4.** Study inquiries. Sources of referral to the study logged by the study team as reported by the participants

Source	Before CE	After CE	Total
*Facebook Ads*	0	164	164
*Direct Mailing*	0	127	127
*Flyer*	15	41	56
*Website*	16	21	37
*Physician Referral*	1	11	12
*Center Watch*	7	2	9
*Friend/Relative*	0	5	5
*Research Match*	4	2	6
*Clinicaltrials.gov*	3	2	5
*Clinicalconnections.com*	0	1	1
*Community talk*	0	1	1
*Google Ads*	0	0	0
*Other/Unknown*	8	53	61
**Total**	**56**	**425**	**481**

**Figure 3.**
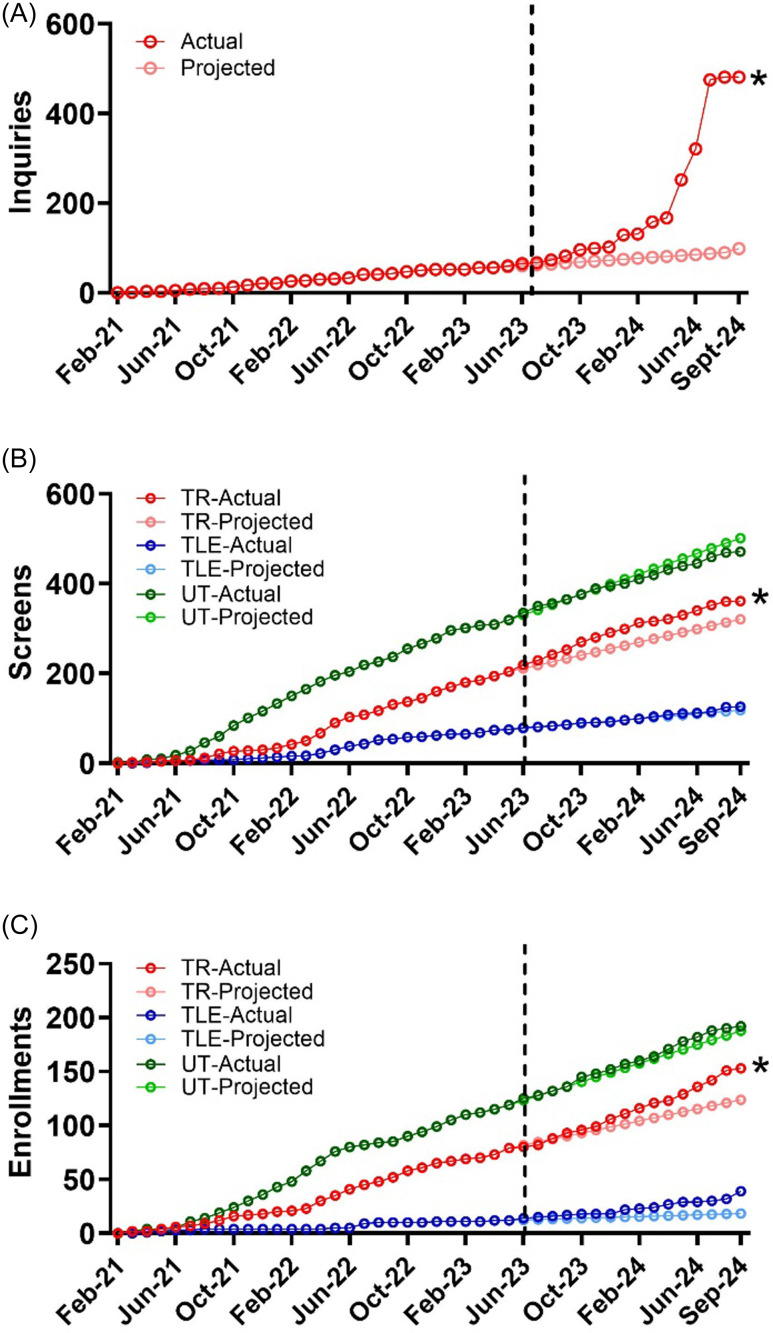
Actual vs projected inquiries, screenings and enrollments. Line graph shows graphs of projected vs actual numbers by month throughout the study timeline for inquiries (A) screenings (B) and enrollments (C). *, *P* < 0.05, significantly different from projected.

Projected inquiries without community engagement based on monthly inquiry community engagement were 90; the actual number of inquiries after community engagement of 481 was significantly greater (760% increase, *t* = 2.41, *p* = 0.018) (Figure 3a). In addition to inquiries to the study team, physical therapists from TR and TLE clinics noted an increase in inquiries particularly after direct mail campaigns.

**Website traffic:** Website traffic prior to community engagement averaged 131 visits per month with a range between 84 and 317. After community engagement, website traffic remained similar until we began Facebook and Google advertisement campaigns. Between June 2024 and August 2024, traffic increased to an average of 9,576 visits per month. Most of the increases came from a direct link (6129) or social media link (2,913). The increased website traffic originated in the areas targeted by our advertisements. We analyzed the traffic from the states we targeted with social medial ads for 5 months before starting ads (Nov 2023-March 2024) and the last 5 months of the study (April 2024-August 2024). Prior to starting ads we had 516 visits (75 rural; 441 urban) from 66 communities within our 7 recruiting states. In the last 5 months, we had 19,085 (9840 rural; 9245 urban) visits from 565 communities showing a greater overall number of visits, greater proportion of rural to urban visits, and a greater number of communities reached.

**Figure 4.**
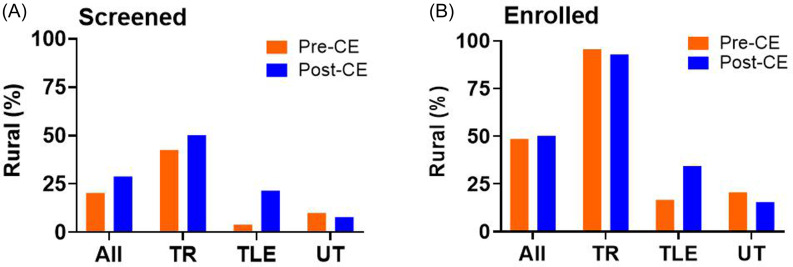
Percentage of rural participants before and after community engagement. The percentage of rural residents screened (A) and enrolled (B) before and after implementation of community engagement strategies (CE) for all clinics (All), TR = targeted rural ; TLE = targeted low enrolling and UT = untargeted clinics ; nearly all TR clinics enrolled rural residents while UT clinics enrolled the least rural residents. TLE clinics showed an increase in screening and enrollment of rural residents.

**Screening:** The timeline of screenings for all clinics (Figure 2d,e) shows a small increase in monthly screening in February 2024 coincides with the direct mail campaign implemented late January 2024. The small increase in screening in July and August 2024 coincides with Facebook ad campaigns active in the months of June, July, and August 2024, as well as direct mail campaigns implemented in the last week of June 2024 and first week of August 2024. The proportion of patients screened that lived in a rural area increased from 20.4 % (122/598) to 28.3% (102/360) across all clinics after implementation of community engagement. Although TLE clinics did not increase screening overall (Supplemental Figure 2), there was an increase in proportion of rural residents screened (Figure 4a).

Community engagement strategies significantly increased screening over expected values for the TR group (*n* = 361 vs 321, 12% increase, *t* = 3.13. *p* = 0.003) but not the TLE group (*n* = 126 vs 118, 6% increase, *t* = 1.14, *p* = 0.27), or the UT group (*n* = 471 vs 501, 6% decrease; *t* = -1.6, *p* = 0.06) (Figure 3b).

**Enrollment:** The timeline of enrollments by month is shown in Figure 2e. Average monthly enrollments across all clinics increased from 9.3 to 12.9 (Figure 2f) with increases occurring only in the TR and TLE groups (Supplemental Figure 2). Across all clinics, the percentage of participants enrolled that lived in a rural area increased from 46.7% before to 52.9% after implementation of community engagement strategies (Figure 4b) with increases occurring in the TLE group. Interestingly, 215 of 918 patients that passed screening lived in a rural area; 214 (99%) of the 215 enrolled in the study. In contrast, 703 of the 918 patients passed screening lived in urban communities; 224 (32%) of the 703 enrolled in the study. The difference between the rate of enrollment between rural and urban residents was significant (c^2^ = 140.3, *p* < 0.0001).

Community engagement strategies significantly increased enrollment over expected values for the TR (*n* = 175 vs 124, 23% increase, *t* = 3.14, *p* = 0.003) and TLE groups (*n* = 39 vs 18, 107% increase, *t* = 2.53, *p* = 0.01) but not for the UT group (*n* = 192 vs 187, 2.7% increase; *t* = 0.20, *p* = 0.42) (Figure 3c). Overall, this represents an additional 82 enrolled participants in all groups above projected enrollment.

## Discussion

The current study implemented a variety of community engagement techniques in both rural and urban communities to enhance enrollment in rural communities and low-enrolling clinics. Successful strategies included partnerships with local physical therapy clinics, individualized flyers hung in local communities, social media advertisements, and postal mailing outreach. Other methods were less successful, including outreach to local physicians and other healthcare providers. The combination of all methods resulted in increases in screening and enrollment beyond expected projections in the TR and TLE groups, but not the UT group, suggesting effectiveness of targeted methods. Community engagement strategies also improve communication and involvement of participating clinics within the study.

### Successful techniques and facilitators

Previous research has identified insufficient awareness of clinical trials as a primary barrier to underrepresented populations’ participation in research, particularly in rural areas, where limited access to information and exposure to healthcare research opportunities make it difficult to foster widespread engagement [22,31,33,40]. The current study found several techniques were highly successful in increasing awareness of the study, but their effectiveness varied across communities. In some areas, individualized flyers displayed at local community sites proved highly effective, whereas in others, direct mail campaigns or social media advertisements had a greater impact. Indeed, prior studies indicate that an individualized approach to community engagement is necessary to enhance research enrollment [34,35,38,41]. Direct mailing and Facebook ads have been reported by others as a useful tool to increase awareness of clinical trials in both rural and urban settings (42-44). While the majority of our Facebook and direct mailing targeted only TR clinics, we also targeted rural communities close to urban clinics (e.g., Fargo, ND) to enhance rural enrollment. Similar to the current study, individualized flyers in local communities were effective to varying degrees in a variety of studies [36,45]. As suggested by others, direct mailings require a greater financial cost than other methods like Facebook ads or hanging flyers [42–44]. Thus, the choice of community engagement technique may depend on a variety of factors including clinic location, cost and staff availability.

### Partnerships as facilitators of successful CE/recruitment strategies

Partnerships with key stakeholders of our research - regional physical therapy healthcare system and their local physical therapists - were essential to the success of community engagement techniques, aligning with previous research [34–36,38,41]. Systematic reviews highlight those partnerships with clinics, local clinicians, and the broader community are crucial for successful recruitment, especially in rural and underserved populations [20,32]. Although community engagement techniques were only formally implemented midway through the recruitment period, the study team had previously built strong relationships within each healthcare system at multiple levels (e.g., clinicians, directors, physician liaisons) during the planning and implementation of FM-TIPS. CE coordinators leveraged these existing connections – particularly with physical therapists local to the rural communities – to effectively design and implement engagement strategies specific to each community. Ongoing communication with local physical therapists facilitated our ability to tailor each strategy for individual communities and to refine each strategy over time. Prior research has shown that collaboration with key community members increases the effectiveness of recruitment strategies [20].

### Secondary effects/community

It is clear that no one method alone increases enrollment in all clinics, but rather combining multiple methods are necessary to enhance enrollment. This is consistent with a number of other community-based clinical trials that suggest the importance of a multi-faceted approach [34,36,38,43,46]. Previous literature identified several promising strategies to recruit underrepresented populations; however, none have emerged as a singularly effective recruitment strategy. Noted as a limitation in many studies includes the inability to quantify specific effects of any single recruitment strategy.

The success of the community engagement techniques may be in part due to secondary effects of increasing engagement of trusted healthcare professionals, particularly in rural communities. Indeed, the current study agrees with previous research findings that engaging participating clinicians in the research process impacts enrollment beyond any individual recruitment method [42]. While many authors agree that a multi-faceted approach is needed, not all report on the importance of partnerships with local health care providers. Specifically, in rural and underserved communities, it seems that partnerships with medical professionals that develop rapport with their patients through multiple interactions and individualized education are highly beneficial [34,36–38]. For example, Kim 2021 attributes their success in recruiting rural residents to their partnerships with local pharmacists who were accessible and personable to patients. In contrast, results from non-rural environments agree that a multi-method approach is necessary to meet recruitment targets, however, the authors do not attribute their success to their partnerships with medical doctors [42,43]. Thus, indirect effects of community engagement, are important components for recruitment in a rural community.

### Barriers/challenges

Patient mistrust in the healthcare system is a barrier to underserved populations seeking treatment and participating in clinical trials [8,22,23,40]. Collaboration with key community members helps develop trust between the community and researchers [20], and patients are more likely to trust medical professionals that spend ample time providing individualized education on their health concerns. For example, Bailey 2004 was able to successfully recruit patients who felt disenfranchized by the medical system by employing a nurse practitioner whose objective to recruit patients was secondary to providing patient education. In the current study, local physical therapists engaged potential participants as part of their broader treatment plan for the patient, supporting evidence of the impact of providing individualized care and education during or as part of the recruitment process.

The burden of travel has been extensively reported as a barrier to rural populations’ participation in clinical research [31,33]. Indeed, a recent study found that rural residents travel further to participate in clinical trials than non-rural residents [18]. The current study addressed this barrier by partnering with local physical therapy clinics (rather than regionalized academic medical centers) and found that rural participants did not travel father than non-rural participants. Interestingly, in the current study, when rural residents passed screening, nearly all enrolled in the study (99%), showing their willingness to participate in research. This is supported by previous research that found rural residents were significantly more likely to complete a digital pain management program than urban residents [47].

A lack of awareness of ongoing clinical trials amongst providers poses a significant barrier to participation in both rural and urban communities [22,31–33]. A systematic review identified that impersonal attempts to engage providers are ineffective and that a prior connection is the primary facilitator of provider engagement [32]. Uniquely, we partnered with each physical therapy clinic to identify referring providers and develop strategies to increase provider awareness of the study. Letters and informational packets were mailed, hand-delivered by the study team, and in some cases these letters and packets were delivered by the physical therapy clinicians themselves. In line with previous research, insufficient infrastructure for communication between researchers and providers posed significant barriers in our attempts to engage providers in informational sessions about the study [41]. However, the letters and information packets did result in an increase in referrals to our participating clinics.

As found by others, community engagement and recruitment in pragmatic clinical trials requires significant effort and resources [32,41]. We had dedicated staff that were trained in community engagement methods to implement these methods. Further, some community engagement techniques are more costly than others and thus a study could choose which methods to implement based on budget. However, a multimodal and individualized approach is the most effective to enhance recruitment. We recommend budgeting community engagement methods from the beginning of the study to maximize engagement and enrollment.

## Limitations

One of the limitations to community engagement is the time and cost to implement. To effectively perform community engagement for 16 targeted clinics in this study required two full-time individuals who were willing to regularly travel to our rural communities. Another limitation for this study was the challenge of accurately determining where a potential participant heard about the study. We attempted to track through self-report, yet it was evident that our survey was often misinterpreted or not filled out. Further, it is impossible to directly measure the indirect effects of visits to clinics and communities, media outreach through radio and local newsletters, and participation in community events. We believe building community rapport building contributed to the willingness of potential participants to participate in the research itself, and the continued engagement of enrolling clinics. Future studies could measure awareness in the community before and after implementation using both quantitative survey approaches and qualitative approaches to better determine the impact of recruitment efforts [40,48,49].

## Conclusions

Implementation of multiple successful community engagement strategies played a critical role in increasing participant enrollment within the study timeframe. A multi-pronged and flexible approach that built and capitalized on strong relationships with community clinics was essential to improve the screening and enrollment of individuals living in rural environments. Community engagement efforts benefit both patients and clinicians to increase their knowledge and access to advances in healthcare research. These findings contribute to a growing body of research demonstrating that pragmatic trials, when combined with strong community engagement, can help bridge healthcare disparities in rural populations.

## Supporting information

10.1017/cts.2025.10103.sm001 Vance et al. supplementary material Vance et al. supplementary material

## Supplementary material

The supplementary material for this article can be found at https://doi.org/10.1017/cts.2025.10103.
