# Supplementary material for: Community engagement strategies improve recruitment and enrollment in a pragmatic clinical trial
Source: J Clin Transl Sci. 2025 Jul 24;9(1):e184. doi: 10.1017/cts.2025.10103 (PMC12444702; doi:10.1017/cts.2025.10103)

**Supplementary Material**

**Supplemental Figure 1.** Examples of A. flyers, B. direct mail postcards and C. Facebook ads.

**Supplemental Figure 2. Average screens and enrollments before and after implementation of community engagement in each group.** Bar graphs show the average screens per month for A. Targeted rural clinics, B. Low enrolling clinics, and C. Untargeted clinics, and enrollments for D. Targeted rural, E. Low Enrolling clinics, and F. Untargeted clinics.

**Supplemental Table 1. Demographics of participants separated by clinic.** Table 1 represents the demographic data from participants who enrolled and completed baseline homework. *Single=divorced, never married, widowed or separated; **With spouse=married or domestic partner; ^+^*Asian/Pacific Islander/Native Hawaiian/American Indian/Alaska Native*

| **Characteristics** | | **Targeted Rural Clinics (N=153)** | **Targeted Low Enrolling Clinics (N=39)** | **Untargeted Clinics (N=192)** | **Total (N=384)** | **P-Value** |
| --- | --- | --- | --- | --- | --- | --- |
| **Age (years)** | *Mean (SD)* | 56.2 (14.3) | 56.2 (13.3) | 50.3 (15.6) | 53.2 (15.2) | **0.0007** |
|  | *Missing* | 3 | 0 | 0 | 3 |  |
| **Sex** | *Male* | 8 (5%) | 4 (10%) | 14 (7%) | 26 (7%) | 0.49 |
|  | *Female* | 142 (93%) | 34 (87%) | 175 (91%) | 351 (91%) |  |
|  | *Other//Not reported/Missing* | 3 (2%) | 1 (3%) | 3 (2%) | 7 (2%) |  |
| **Household Income** | *Less than $10,000* | 10 (7%) | 1 (3%) | 21 (11%) | 32 (8%) | 0.54 |
|  | *$10,000-34,999* | 44 (29%) | 9 (23%) | 48 (25%) | 101 (26%) |  |
|  | *$35,000-74,999* | 40 (26%) | 12 (31%) | 46 (24%) | 98 (26%) |  |
|  | *$75,000 or more* | 39 (25%) | 9 (23%) | 51 (27%) | 99 (26%) |  |
|  | *Not reported/Missing* | 20 (13%) | 8 (21%) | 26 (14%) | 54 (14%) |  |
| **Marital Status** | *Single** | 48 (31%) | 9 (23%) | 78 (41%) | 135 (35%) | **0.029** |
|  | *With spouse*** | 102 (67%) | 30 (77%) | 106 (55%) | 238 (62%) |  |
|  | *Not reported/Missing* | 3 (2%) | 0 (0%) | 8 (4%) | 11 (3%) |  |
| **Ethnicity** | *Hispanic or Latino* | 5 (3%) | 1 (3%) | 20 (10%) | 26 (7%) | **0.02** |
|  | *Not Hispanic or Latino* | 132 (86%) | 36 (92%) | 158 (82%) | 326 (85%) |  |
|  | *Unknown/Not Reported/Missing* | 16 (10%) | 2 (5%) | 14 (7%) | 32 (8%) |  |
| **Race** | *White* | 141 (92%) | 34 (87%) | 140 (73%) | 315 (82%) | **<0.000** |
|  | *Black* | 2 (1%) | 2 (5%) | 28 (15%) | 32 (8%) |  |
|  | *Other^+^* | 2 (1%) | 1 (3%) | 5 (3%) | 8 (2%) |  |
|  | *Multiracial* | 3 (2%) | 1 (3%) | 3 (2%) | 7 (2%) |  |
|  | *Unknown/Not Reported/Missing* | 5 (3%) | 1 (3%) | 16 (8%) | 22 (6%) |  |
| **Education Status** | *High school or less* | 59 (39%) | 22 (56%) | 68 (35%) | 149 (39%) | **0.01** |
|  | *Some college/ vocational/*  *technical degree* | 53 (35%) | 12 (31%) | 50 (26%) | 115 (30%) |  |
|  | *College and above* | 37 (24%) | 5 (13%) | 67 (35%) | 109 (28%) |  |
|  | *Not Reported/Missing* | 4 (3%) | 0 (0%) | 7 (4%) | 11 (3%) |  |
| **Working Status** | *Working* | 52 (34%) | 18 (46%) | 90 (47%) | 160 (42%) | 0.1036 |
|  | *Unemployed* | 91 (59%) | 21 (54%) | 98 (51%) | 210 (55%) |  |
|  | *Removed* | 10 (7%) | 0 (0%) | 4 (2%) | 14 (4%) |  |
| **Distance to clinic (miles)** | *Mean (SD)* | 9.4 (12.0) | 9.5 (10.6) | 8.6 (12.3) | 9.0 (12.0) |  |

**Supplemental Table 2. Flyers.** Dates of visits to communities where flyers were hung for our Targeted Rural (TR) and Targeted Low Enrolling (TLE) groups and the number of clinics targeted each month are shown in the table.

| **Flyer Date**​ | **Clinic Target Status**​ | **# of Clinics**​ |
| --- | --- | --- |
| **Jun-23**​ | TR ​ | 6 ​ |
|  | TLE ​ | 2 ​ |
| **Jul-23**​ | TR ​ | 5 ​ |
|  | TLE ​ | 0 ​ |
| **Aug-23**​ | TR ​ | 2 ​ |
|  | TLE ​ | 0 ​ |
| **Sep-23**​ | TR ​ | 9 ​ |
|  | TLE ​ | 0 ​ |
| **Nov-23**​ | TR ​ | 5 ​ |
|  | TLE ​ | 4 ​ |
| **Jan-24**​ | TR ​ | 1 ​ |
|  | TLE ​ | 2 ​ |
| **Feb-24**​ | TR ​ | 9 ​ |
|  | TLE ​ | 0 ​ |
| **Mar-24**​ | TR ​ | 4 ​ |
|  | TLE ​ | 0 ​ |
| **Apr-24**​ | TR ​ | 1 ​ |
|  | TLE ​ | 3 ​ |
| **Jul-24**​ | TR ​ | 9 ​ |
|  | TLE ​ | 1 ​ |
| **Aug-24**​ | TR ​ | 9 ​ |
|  | TLE ​ | 3 ​ |

Supplemental Figure 1


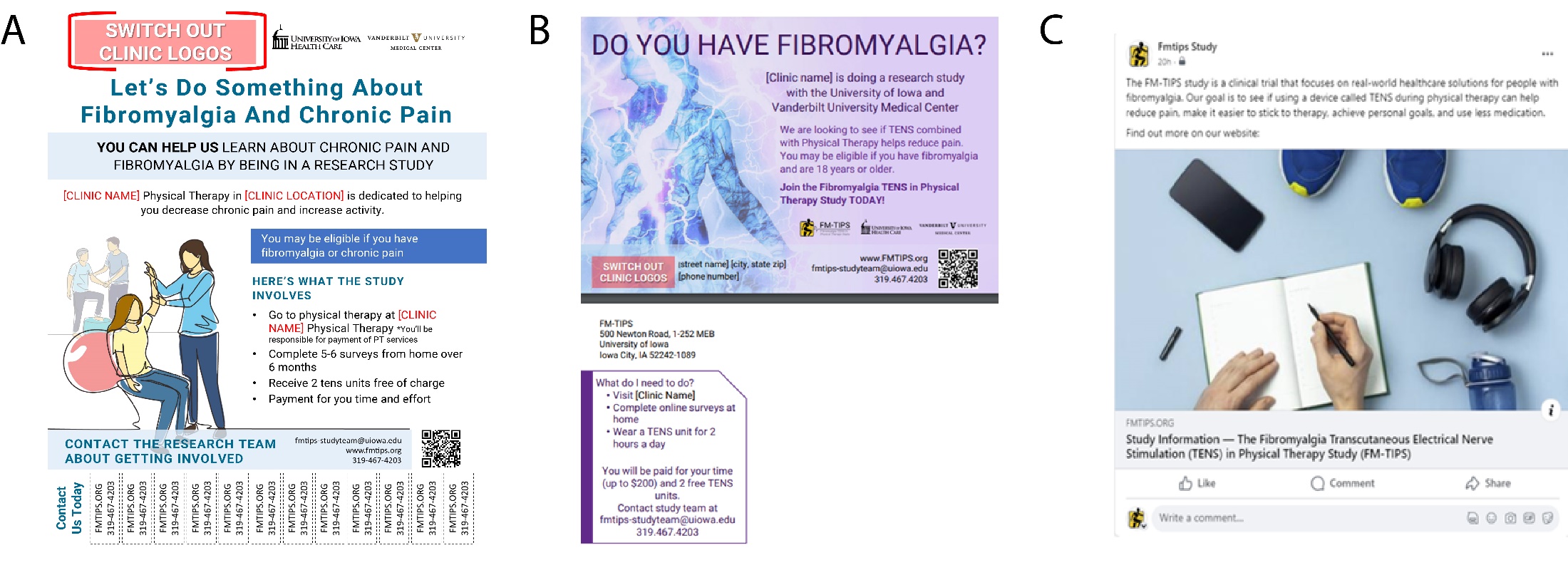


Supplemental Figure 2


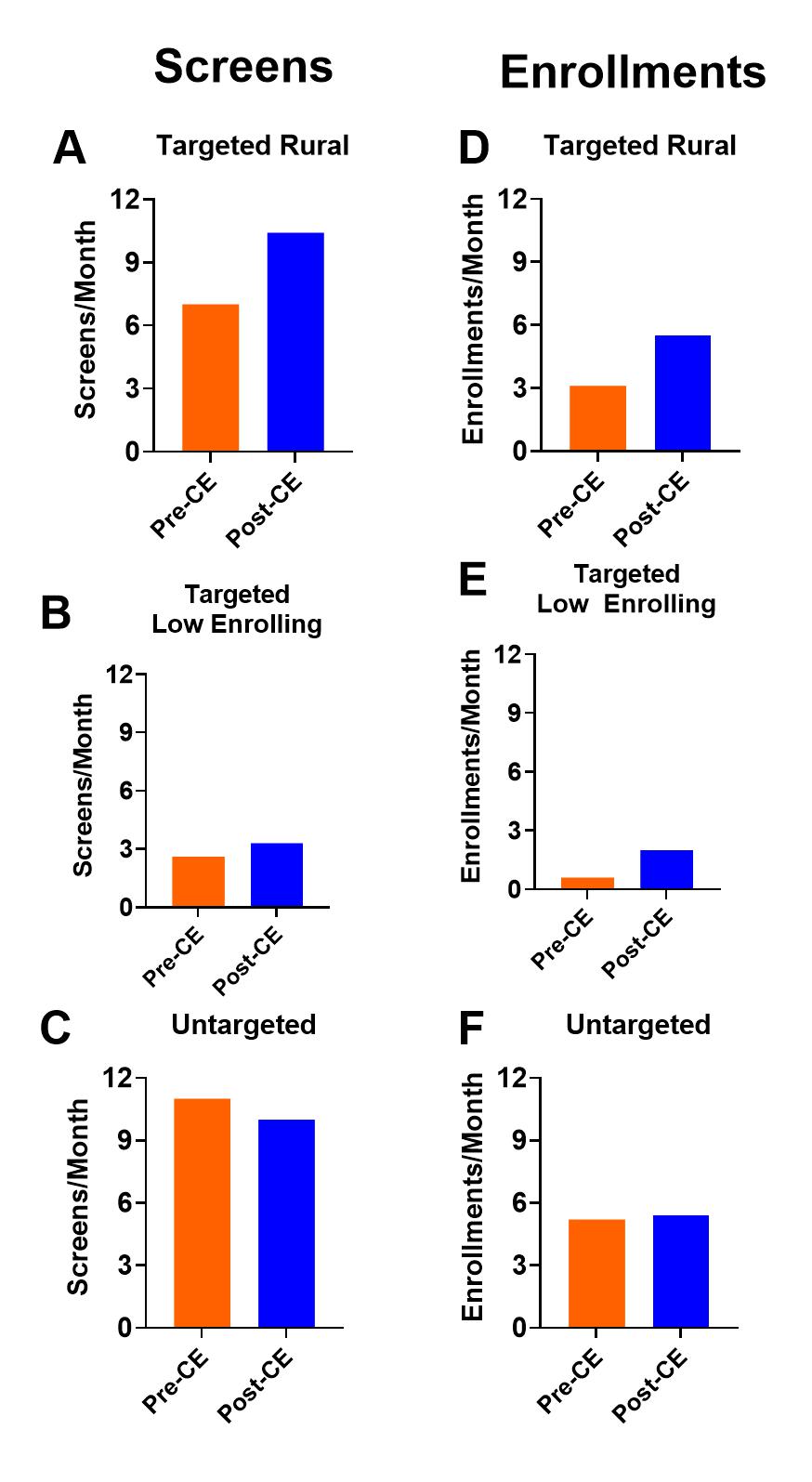

Supplement: Vance et al. supplementary material [file S2059866125101039sup001.docx]
